# Supplementary figures and images for: Fact and Fiction about 1%: Next Generation Sequencing and the Detection of Minor Drug Resistant Variants in HIV-1 Populations with and without Unique Molecular Identifiers
Source: Viruses. 2020 Aug 4;12(8):850. doi: 10.3390/v12080850 (PMC7472098; doi:10.3390/v12080850)

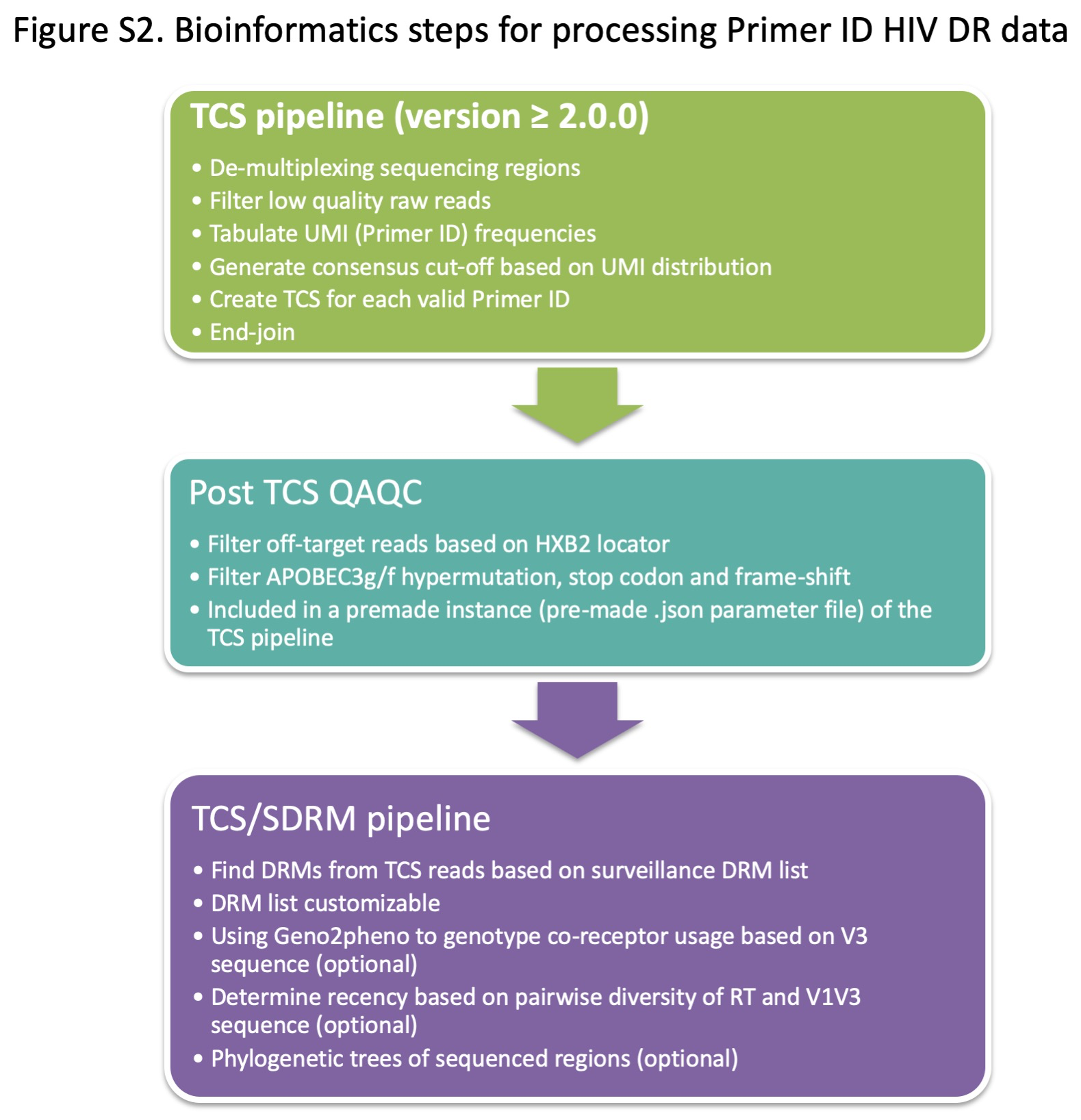

Supplement: Supplementary file 1 [file viruses-12-00850-s001.zip › figure s1 and s2/Figure S2.png]

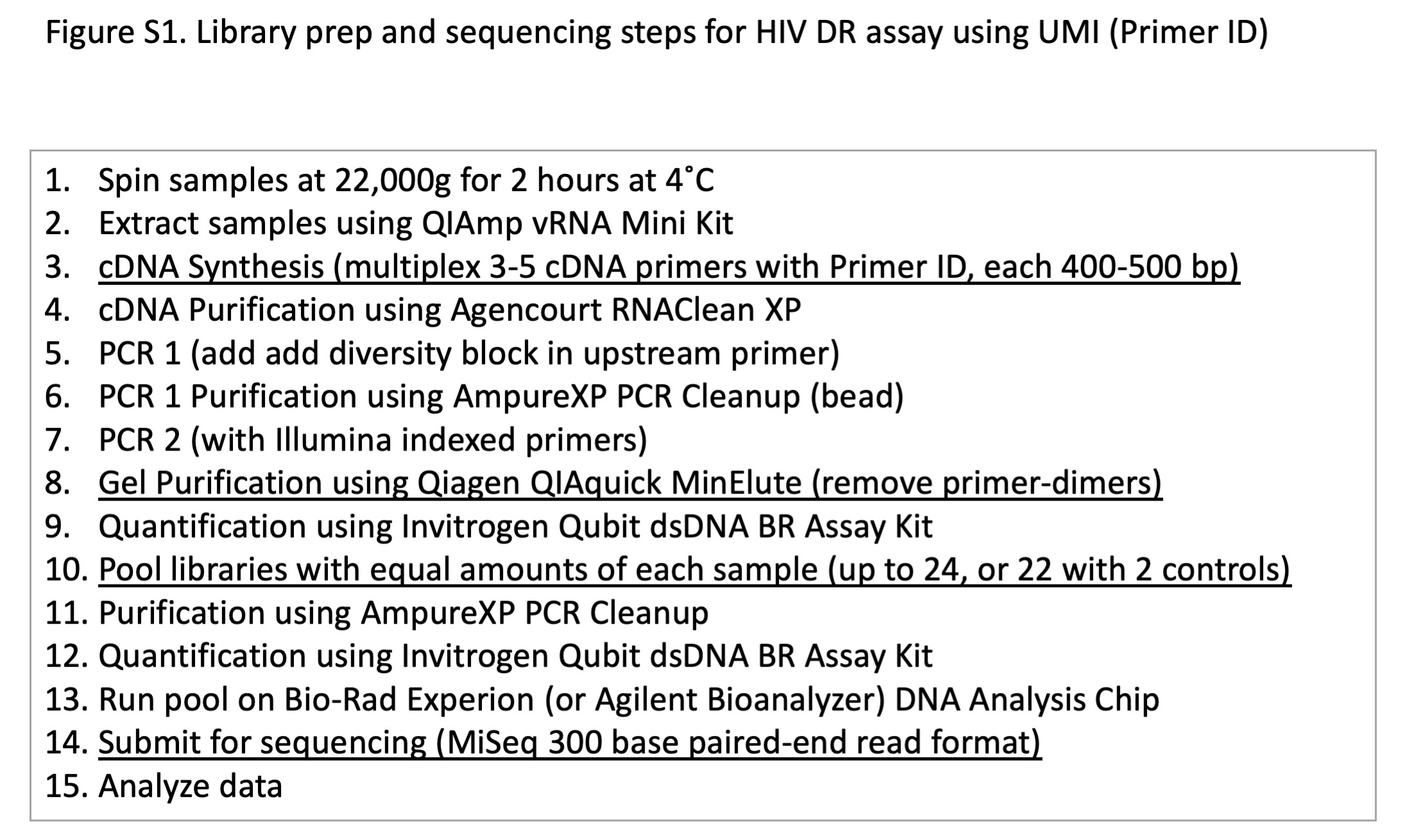

Supplement: Supplementary file 1 [file viruses-12-00850-s001.zip › figure s1 and s2/Figure S1.png]
